# The impact of perineural invasion on prognosis in esophageal cancer patients after surgery: a systematic review and meta-analysis

**DOI:** 10.3389/fonc.2025.1629335

**Published:** 2025-09-10

**Authors:** Jicheng Xiong, Shuoming Liang, Simiao Lu, Hainan Chen, Ziwei Wang, Hao Meng, Yi Zhu, Yongtao Han, Xuefeng Leng

**Affiliations:** ^1^ School of Medicine, University of Electronic Science and Technology of China, Chengdu, China; ^2^ School of Clinical Medicine, Chengdu Medical College, Chengdu, China; ^3^ Department of Thoracic Surgery, Sichuan Cancer Hospital & Institute, Sichuan Cancer Center, University of Electronic Science and Technology of China, Chengdu, China; ^4^ Department of Ultrasound, Sichuan Cancer Hospital & Institute, Sichuan Cancer Center, University of Electronic Science and Technology of China, Chengdu, China

**Keywords:** esophageal cancer, perineural invasion, prognosis, distant metastasis, recurrence

## Abstract

**Objective:**

This study aims to update the prognostic value of perineural invasion(PNI) in various subgroups of esophageal cancer patients.

**Methods:**

We searched databases including PubMed, Scopus, Wiley, Web of Science, and Embase for full-text articles published in English on esophageal cancer related to PNI. The search was conducted up to January 1, 2024. We summarized the hazard ratios (HR) and 95% confidence intervals (CI) for overall survival (OS), disease-free survival (DFS), as well as recurrence and metastasis, to assess the prognostic value of PNI in patients with esophageal cancer.

**Results:**

A total of 38 eligible studies were ultimately included. Thirty-two studies, encompassing a total of 7157 patients, reported the correlation between PNI and OS. The results indicated that PNI is significantly associated with poor OS in esophageal cancer patients (HR = 1.54, 95% CI: 1.41-1.68, P < 0.00001). Eleven studies, including a total of 2224 patients, reported the correlation between PNI and DFS. These studies found that PNI is significantly associated with poor DFS (HR = 1.43, 95% CI: 1.25-1.62, P < 0.00001). Three studies, including a total of 1125 patients, reported no correlation between PNI and recurrence (HR = 1.17, 95% CI: 0.62-2.18, P = 0.63). Two studies, including a total of 556 patients, reported a correlation between PNI and distant metastasis (HR = 2.19, 95% CI: 1.02-4.73, P = 0.04). Further subgroup analysis revealed that PNI is an independent prognostic factor for esophageal squamous cell carcinoma (ESCC) (OS: HR=1.62, 95%CI: 1.35-1.94, P<0.00001; DFS: HR=1.28, 95%CI: 1.03-1.59, P=0.03); however, in esophageal adenocarcinoma, PNI is not associated with OS or DFS (OS: HR=1.23, 95%CI: 1.00-1.53, P=0.05; DFS: HR=1.65, 95%CI: 0.95-2.87, P=0.08). PNI positivity is associated with unfavorable outcomes, irrespective of neoadjuvant therapy receipt. In the non-Asian subgroup, PNI is not statistically significant for poor DFS prognosis.

**Conclusion:**

PNI is a histological marker of aggressive disease and can serve as an independent prognostic factor for patients with esophageal cancer. PNI positivity can predict poor outcomes in ESCC, but its role as a prognostic indicator for adenocarcinoma requires further investigation.

## Introduction

Esophageal cancer (EC) is one of the top ten cancers worldwide, with 200,000 new cases occurring annually, half of which are in China (1). Asia is a high-incidence region for esophageal cancer, predominantly esophageal squamous cell carcinoma (ESCC), and the prognosis is poor. With advancements in diagnostic and therapeutic technologies, multidisciplinary treatments centered around esophagectomy have improved the survival rates of EC patients, but the five-year overall survival (OS) rate is still less than 50%. Additionally, while the use of adjuvant radiochemotherapy can reduce the risk of recurrence and metastasis, the toxic side effects on the heart and lungs severely affect the quality of life of patients and can even decrease OS rates. Identifying factors that can accurately predict the prognosis of EC patients to guide clinical management and treatment strategies, provide personalized treatment plans, and minimize the toxic side effects of perioperative radiochemotherapy, thereby improving the quality of life and OS rates of patients, is increasingly valued by clinical practitioners.

The prognosis of EC largely depends on the TNM staging of the disease. Among the factors, whether lymph nodes have metastasized is the most critical indicator for assessing the prognosis of EC; however, more than 40% of patients with negative lymph nodes still experience recurrence or metastasis. Therefore, it is necessary to incorporate additional biological factors to evaluate patient prognosis. Previous studies have shown that in various types of cancer patients, including those with positive lymphatic vessel invasion (LVI) and perineural invasion (PNI) have a significantly poorer prognosis. It is worth noting that in patients with various types of cancer, including EC, the LVI is considered an independent prognostic pathological factor, but the prognostic role of PNI has not reached a consensus. Initially, PNI was considered an ancillary form of lymphatic infiltration, but subsequent studies have indicated that the perineural space lacks lymphatic vessels, thus PNI is a distinct form of micro-invasion (2). A positive PNI usually signifies micro-metastasis, which may lead to incomplete tumor resection, thereby reducing the quality of life for patients and even directly affecting survival rates, as has been reported in other types of cancer (3–11). In recent years, the literature on prognostic factors for EC has frequently mentioned that PNI may impact prognosis (12–14), but some scholars have raised doubts about this (15–18). Therefore, it is necessary to reassess whether a positive PNI can serve as an independent prognostic factor for EC patients in order to accurately guide clinical treatment.

In order to obtain an accurate conclusion on this controversial topic, we conducted a systematic review and meta-analysis to update the prognostic value of PNI in patients with EC. Unlike previous meta-analyses, this study includes disease-free survival (DFS), recurrence, and metastasis into the analysis, and performs subgroup analyses based on pathological type, preoperative neoadjuvant therapy, and race to clarify the prognostic significance of PNI in different subgroups.

## Materials and methods

### Inclusion and exclusion criteria

The inclusion criteria are: 1) Patients who underwent esophagectomy, with pathological confirmation of PNI, and have complete follow-up data; 2) Studies that report the association between PNI and OS, DFS, or other prognostic outcomes (such as recurrence, metastasis); 3) Studies that provide hazard ratios (HR) and 95% confidence intervals (CI) or raw data sufficient to calculate risk ratios. The exclusion criteria are: 1) Patients with a confirmed history of other malignancies; 2) Studies published only as abstracts, reviews, and case reports.

### Sources of information

We searched databases such as PubMed, Scopus, Wiley, Web of Science, and Embase for English full-text literature on PNI in EC. The search was conducted up to January 1, 2024. This article was written following the PRISMA 2020 guidelines (19).

### Search strategy

The search keywords were: “esophageal cancer” and “perineural invasion”. The search was conducted using a combination of MeSH terms and free-text words. For example, the search query in PubMed was: (((((((((“Esophageal Neoplasms”[Mesh]) OR (neoplasm, esophageal)) OR (Esophagus Neoplasm)) OR (neoplasm, esophagus)) OR (Cancer of Esophagus)) OR (Cancer of the Esophagus)) OR (Esophagus Cancer)) OR (Cancer, Esophagus)) OR (Esophageal Cancer)) AND (perineural invasion). Additional filtering criteria included full-text articles published in English regarding human studies.

### Data collection process

**Figure 1:**
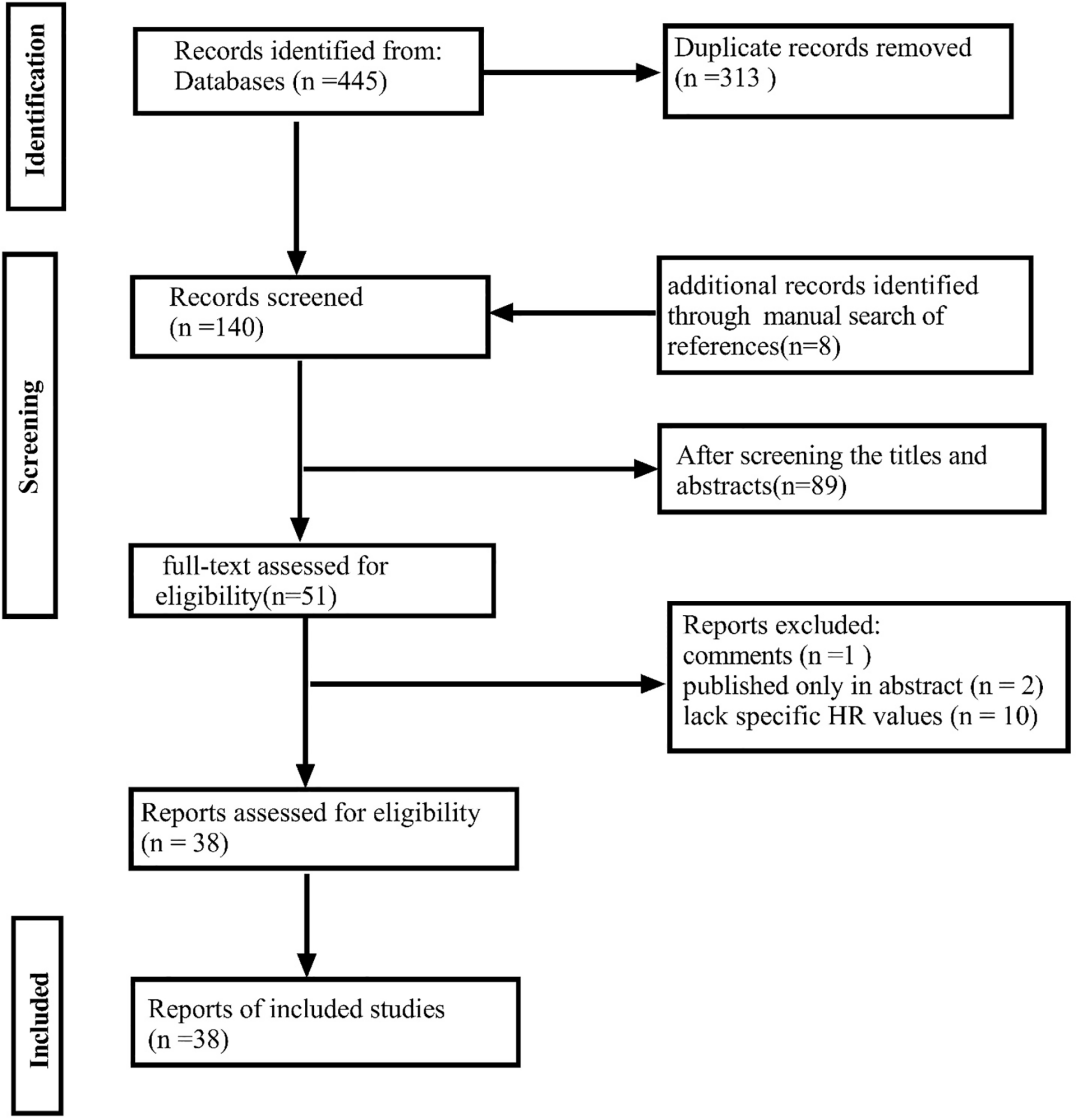
PRISMA 2020 flow diagram illustrating the study selection process for the systematic review and meta-analysis.

After removing duplicate literature, three authors (Xiong, Liang, and Lu) independently reviewed the titles and abstracts to exclude irrelevant literature. Further reading of the full text was conducted to determine eligible studies. Additionally, the three authors manually searched the reference lists of eligible studies to identify potentially eligible research. In case of disagreements among the three authors, discussions were held until a consensus was reached. The detailed screening process is illustrated in **Figure 1**.

### Heterogeneity in PNI assessment

All included studies were retrospective, and no uniform criteria for diagnosing PNI were applied. Among those that did specify their approach, most relied primarily on hematoxylin–eosin (H&E) staining: unequivocal morphologic findings were accepted as definitive, whereas equivocal cases prompted supplementary immunohistochemistry for confirmation; a minority of cases were ultimately adjudicated by experienced pathologists based on integrated histopathologic judgment. Several studies provided no details regarding diagnostic criteria.

### Data extraction

Data was independently extracted by three authors (Xiong, Liang, and Lu) following a standard protocol. The following information was collected from each study: first author, publication year, study design, patient characteristics (including region, sample size, age, gender, PNI positivity rate, etc.), clinical and pathological features (perioperative treatment, TNM staging, pathological grading, pathological type, etc.), prognosis (OS and DFS), and other outcomes (recurrence, metastasis). Hazard ratios (HR) and 95% confidence intervals related to overall survival, disease-free survival, recurrence, and metastasis were extracted from all literature.

### Risk assessment

The Newcastle-Ottawa Scale was used to formally assess the quality of the studies (20), with a score of ≥6 indicating high quality and <3 indicating low quality. Additionally, the Cochrane Bias Tool was employed to evaluate the studies.

### Data synthesis and grouping

Based on the HR values and P values, we calculated the standard error using the inverse variance method. We have strictly limited the primary Meta-analysis to only combining HR from multivariable-adjusted Cox proportional risk models. The need to report other effect sizes (OR) or univariate HR has been excluded from the primary analysis. If the article did not directly provide HR data but provided RR data, HR and RR can be combined, as they represent the same meaning without considering the time factor.

### Statistical analysis

The meta-analysis will be conducted using Review Manager V5.4 software (Copenhagen, The Nordic Cochrane Centre, The Cochrane Collaboration). The heterogeneity of the studies will be assessed using the Q test and I² statistic. If the Q-P value is less than 0.1 and/or I² is greater than 50%, significant heterogeneity is considered to be present, and further analysis will be conducted. If no significant heterogeneity is found, a fixed-effect model will be used to obtain the pooled estimate of HR; otherwise, a random-effects model will be used. The pooled HR values will be plotted in a forest plot, with P < 0.05 considered statistically significant. Funnel plots will be constructed using Chrone to visually assess the publication bias of the included studies. The asymmetry of the funnel plot will be evaluated using Egger’s test.

## Results

### Literature search and study characteristics

A total of 445 potentially eligible studies were identified based on our search strategy. After excluding 313 duplicate studies, 91 studies after title and abstract screening, 1 review and 2 abstract - only studies, and 9 studies due to missing or incomputable HR values, we found 8 more studies by reviewing reference lists. Ultimately, we included 39 retrospective cohort studies published from 2011 to 2023 (15–18, 21–27, 28–54). These studies, with sample sizes ranging from 26 to 794 (median: 174), reported on the correlation between PNI and outcomes. Specifically, 32 studies examined the correlation between PNI and unfavorable OS (15, 16, 18, 23, 25, 28, 32, 33, 35, 36, 38, 40–43, 45, 47–53, 55, 56), 11 focused on PNI and DFS (15, 23, 25, 29, 38, 39, 44, 46, 47, 51, 54), and 5 analyzed the relationship between PNI and recurrence/metastasis (16, 34, 36, 37, 40). The median PNI positivity rate was 25% (8% - 73%). Among the included studies, 18 focused solely on ESCC patients,8 on adenocarcinoma patients (17, 22, 29, 32, 36, 42, 52, 54), and the remaining 13 on mixed or other pathological types. Geographically, 26 studies targeted Asian populations, and 13 non - Asian populations. For specific details, see **Table 1**. (Specific pathological characteristics and details are shown in **Table 1**).

**Table 1:** Baseline characteristics of included cohorts.

Study	Country	physiology	nRCT	N	PNI+ (%)	HR (95% CI)	P-value	Outcome	Study quality
Alcan S et al., 2022 (18)	Turkish	AEC	Yes	50	60	2.155(0.691-6.724)	0.186	OS	7
Mathieu et al., 2023 (19)	Canada	EA	No	103	55	1.18(0.48–2.9)	0.717	DFS	6
Ning ZZ et al., 2015 (53)	China	ESCC	No	243	22	1.832(1.267-2.651)	0.001	OS	8
Chen JW et al., 2014 (45)	China	ESCC	No	433	48	1.374(1.037-1.820)	0.027	OS	8
Wang H et al., 2017 (48)	China	ESCC	*	466	24	1.298(0.863-1.952)	0.210	OS	6
Tu CC et al., 2017 (47)	China	ESCC	Yes	91	16	OS:2.226(1.144–4.331) DFS:1.481(0.693–3.164)	0.019 0.311	OS、DFS	8
Kim H et al., 2021 (46)	Korea	ESCC	*	316	8	1.890(1.088–3.282)	0.024	DFS	6
Xu GH et al., 2017 (44)	China	ESCC	No	302	51	1.506(1.248–1.818)	<0.001	DFS	7
Tsai C et al., 2017 (43)	Taipei	ESCC	No	177	44	1.286(0.830-1.944)	0.26	OS	7
Patel A et al., 2020 (42)	USA	EA	Yes	73	40	0.64(0.74 -2.74)	0.18	OS	7
Guo YN et al., 2020 (41)	China	ESCC	Yes	162	73	1.937(0.974–3.851)	0.059	OS	6
Sheng LM et al., 2015 (40)	China	ESCC	No	148	25	OS:3.56(1.62–7.84) MT: 2.35(1.04–5.29)	0.002 0.039	OS、MT	7
Ma Y et al., 2022 (38)	China	ESCC	No	349	36	OS:1.6(1.2–2.1) DFS:1.4(1.1–1.9)	0.001 0.017	OS、DFS	7
Zhang L et al., 2022 (16)	China	ESCC	No	794	16	OS:0.688(0.448-1.056) RC:0.837(0.551–1.273)	0.087 0.406	OS、RC	7
Zhang WY et al., 2018 (37)	China	ESCC	*	408	*	1.598(1.153–2.214)	0.005	MT	7
Hsu CP et al., 2019 (31)	Taipei	ESCC	*	520	23	2.255(1.431-3.556)	<0.001	OS	7
Hsua P.K et al., 2017 (34)	Taipei	ESCC	Yes	116	16	2.053(0.765–5.506)	0.15	RC	7
Zhou JF et al., 2023 (15)	China	ESCC	Yes	321	18	OS:1.378(0.778-2.442) DFS:1.41(0.843-2.368))	0.271 0.190	OS、DFS	7
Xie CK et al., 2021 (33)	China	ESCC	No	195	*	1.159(0.730–1.838)	0.532	OS	7
Tapias L et al., 2020 (32)	USA	EA	*	196	53	1.09(0.70–1.72)	0.696	OS	7
Hsua P.K et al., 2018 (35)		ESCC	Yes	150	25	4.619(2.492–8.560))	<0.001	OS	8
Su NW et al., 2023 (30)	China	ESCC	Yes	150	*	2.354(1.240-4.467)	0.009	OS	8
Vošmik et al., 2017 (29)	Czech	*	Yes	108	12	OS:2.09(0.85–5.12) DFS:2.03(0.80–5.16)	0.049 0.031	OS、DFS	7
Hardy K et al., 2023 (17)	UK	EA	No	172	28	1.115(0.603–2.061)	0.729	OS	6
Griffin N et al., 2020 (24)	Australia	*	*	260	12	2.45(1.03-5.84)	0.04	OS	6
Tanke J et al., 2022 (22)	Canada	EA	Yes	167	*	2.51(1.03–6.08)	0.048	OS	6
Tanaka A et al., 1998 (27)	Japan	*	No	104	46	2.34 (*)	0.0198	OS	6
Sun YH et al., 2015 (21)	China	CT	*	26	19	4.986(1.491–16.675)	0.009	OS	6
KIM T et al., 2011 (26)	USA	AEC	No	266	11	2.44(0.48-4.40)	<0.0001	OS	6
Rong LL et al., 2019 (39)	China	ESCC	No	378	33	OS:1.328(0.999-1.764) DFS:1.325(1.012-1.736)	0.051 0.041	OS、DFS	8
Singhi A et al., 2015 (52)	USA	EA	*	205	46	1.12(0.78-1.60)	0.547	OS	6
Wang H et al., 2017 (48)	China	ESCC	*	466	24	1.298(0.863–1.952)	0.210	OS	6
Merritt R.E et al., 2020 (36)	USA	EA	Yes	215	*	OS:1.14(0.93-2.22) RC:1.38(0.92-2.07)	0.101 0.116	OS、RC	7
Noble F et al., 2013 (50)	UK	*	*	246	14	1.980(1.218–3.218)	0.006	OS	6
Hsieh C et al., 2016 (23)	Taipei	ESCC	No	81	30	OS:1.79(1.01-3.15) DFS:0.95(0.49-1.85)	0.89 0.201	OS、DFS	7
Lee HK et al., 2020 (51)	Korea	ESCC	No	64	20	OS:1.435(0.646–3.188) DFS:1.069(0.449–2.544)	0.376 0.880	OS、DFS	8
Peng HJ et al., 2021 (49)	China	ESCC	*	121	10	0.929(0.371–2.327)	0.875	OS	6
Zeng YZ et al., 2021 (25)	China	*	*	97	10	OS:1.421(0.599–3.373) DFS:1.683(0.755–3.750)	0.203 0.426	OS、DFS	6

EA, esophageal adenocarcinoma; ESCC, esophageal squamous cell carcinoma; esophageal colloid tumor; OS, overall survival; DFS, disease-free survival; RE, recurrence of esophageal cancer; ME, metastasis of esophageal cancer; *: unknown.

### Meta-analysis result

**Figure 2:**
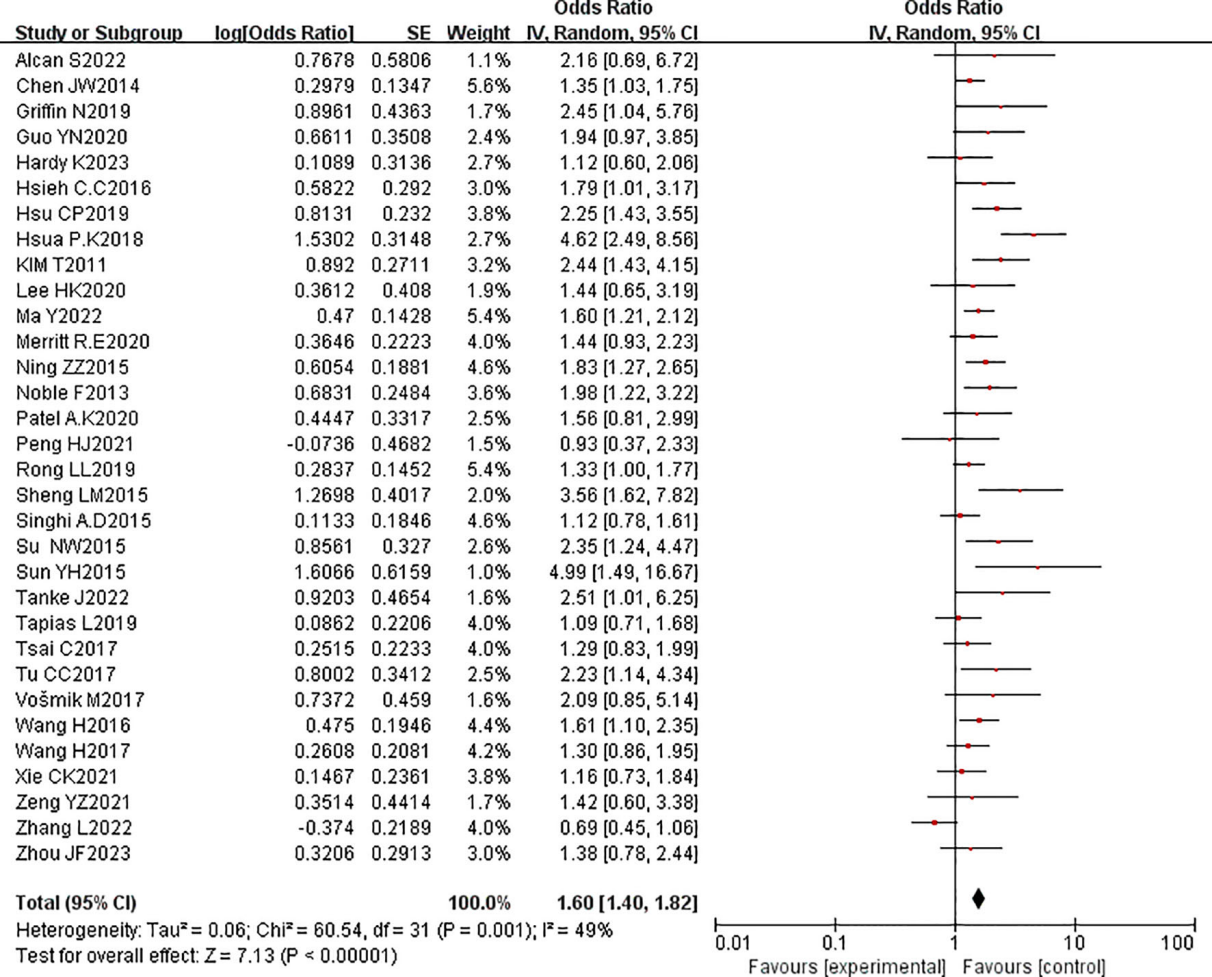
Forest plot of the pooled hazard ratio (HR) for the association between perineural invasion (PNI) and overall survival (OS) in esophageal cancer patients.

**Figure 3:**
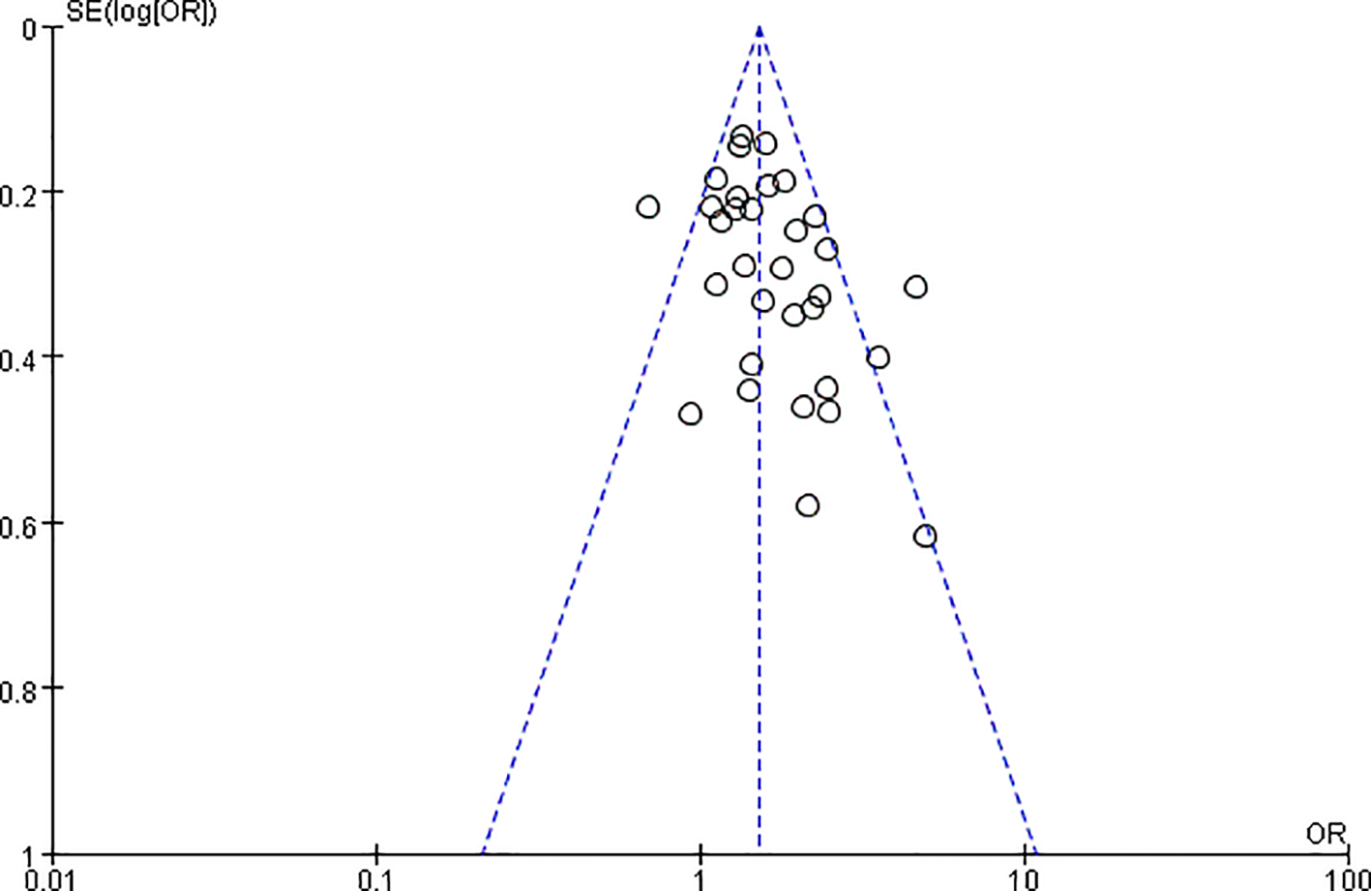
Funnel plot for the assessment of publication bias in the meta-analysis of overall survival (corresponding to **Figure 2**).

In a meta-analysis of 32 studies involving 7,157 patients with esophageal cancer, the correlation between PNI and poor OS was reported. The pooled HR indicated that PNI positivity was associated with adverse OS after esophageal cancer surgery (HR = 1.54, 95% CI: 1.41-1.68, P < 0.00001). Due to significant heterogeneity (P = 0.002, I² = 48%), a random-effects model was employed (**Figure 2**). By sequentially excluding studies, the sources of heterogeneity were identified (studies 39 and 46). After excluding these studies, heterogeneity was significantly reduced (P = 0.25, I² = 14%). A funnel plot intuitively demonstrated the presence of publication bias (**Figure 3**).

**Figure 4:**
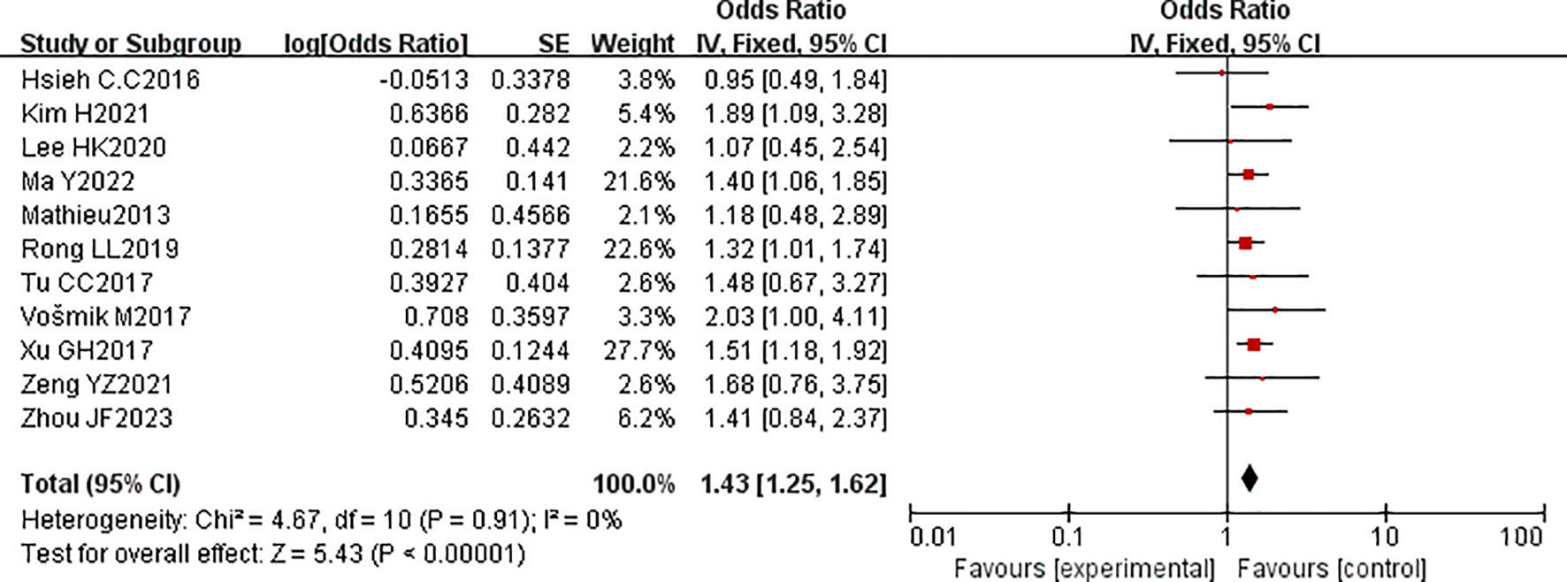
Forest plot of the pooled hazard ratio (HR) for the association between perineural invasion (PNI) and disease-free survival (DFS) in esophageal cancer patients.

**Figure 5:**
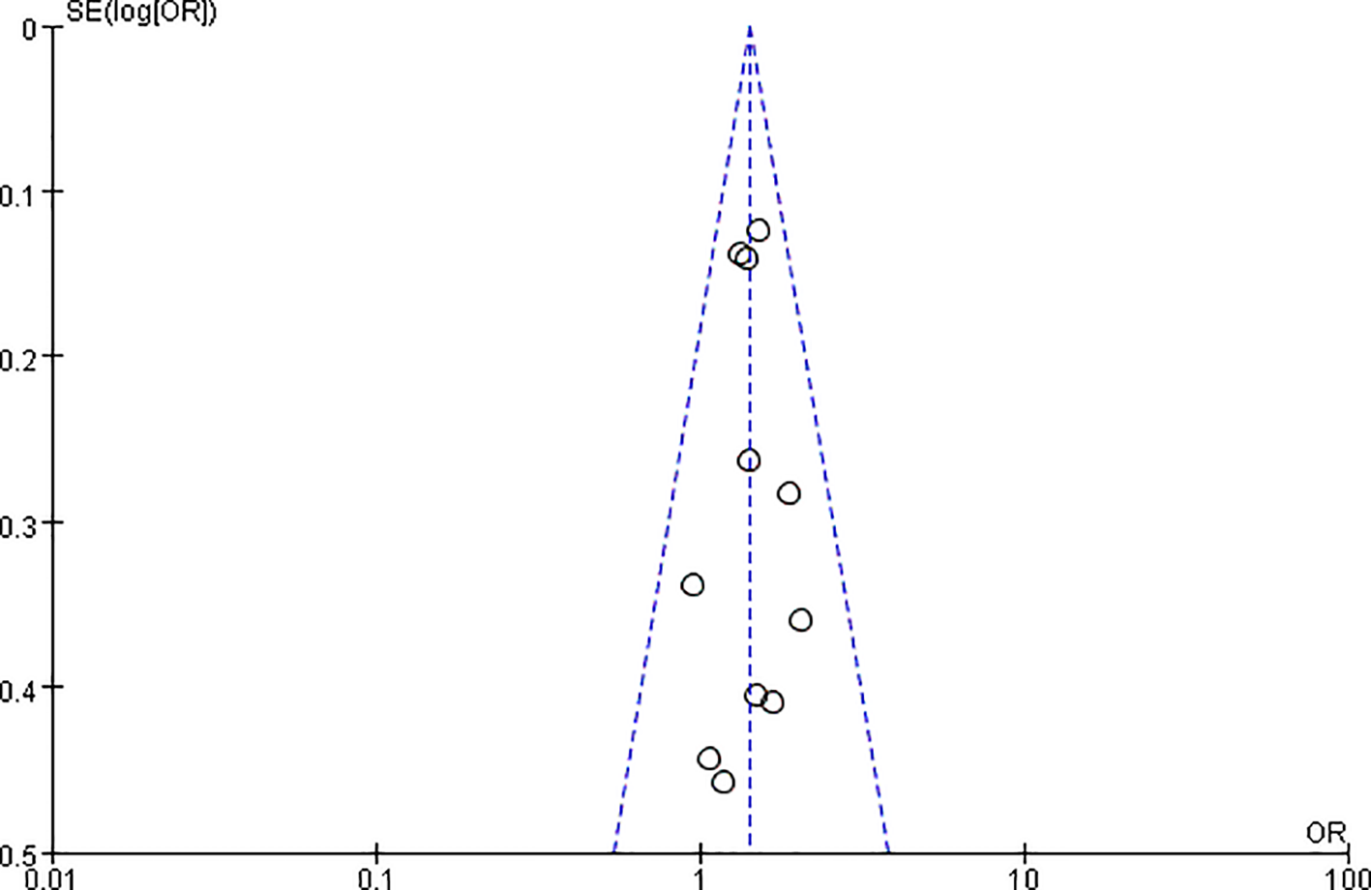
Funnel plot for the assessment of publication bias in the meta-analysis of disease-free survival (corresponding to **Figure 4**).

Eleven studies encompassing 2,224 patients with esophageal cancer reported the correlation between PNI and poor DFS. The pooled HR revealed that PNI is a prognostic factor for adverse DFS after esophageal cancer surgery (HR = 1.43, 95% CI: 1.25-1.62, P < 0.00001). Given the absence of significant heterogeneity (P = 0.91, I² = 0%), a fixed-effects model was applied (**Figure 4**). No evidence of publication bias was detected (**Figure 5**).

**Figure 6:**
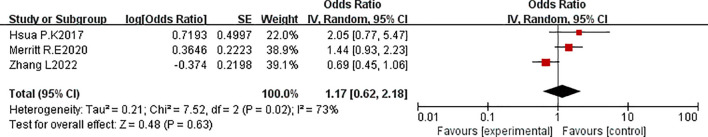
Forest plot of the pooled hazard ratio (HR) for the association between perineural invasion (PNI) and postoperative recurrence.

Three studies involving 1,125 patients with esophageal cancer reported the correlation between PNI and recurrence. The pooled HR showed no statistical significance (HR = 1.17, 95% CI: 0.62-2.18, P = 0.63). Significant heterogeneity was observed (P = 0.02, I² = 73%), necessitating the use of a random-effects model. No evidence of publication bias was found (**Figure 6**).

**Figure 7:**
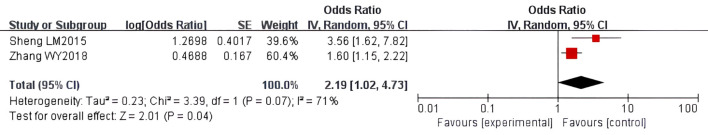
Forest plot of the pooled hazard ratio (HR) for the association between perineural invasion (PNI) and distant metastasis.

Two studies including 556 patients with esophageal cancer reported the correlation between PNI and distant metastasis. The pooled HR indicated that PNI positivity was associated with distant metastasis (HR = 2.19, 95% CI: 1.02-4.73, P = 0.04). Significant heterogeneity was present (P = 0.07, I² = 71%), and a random-effects model was employed. No evidence of publication bias was detected (**Figure 7**).

### Stratified analysis

**Figure 8:**
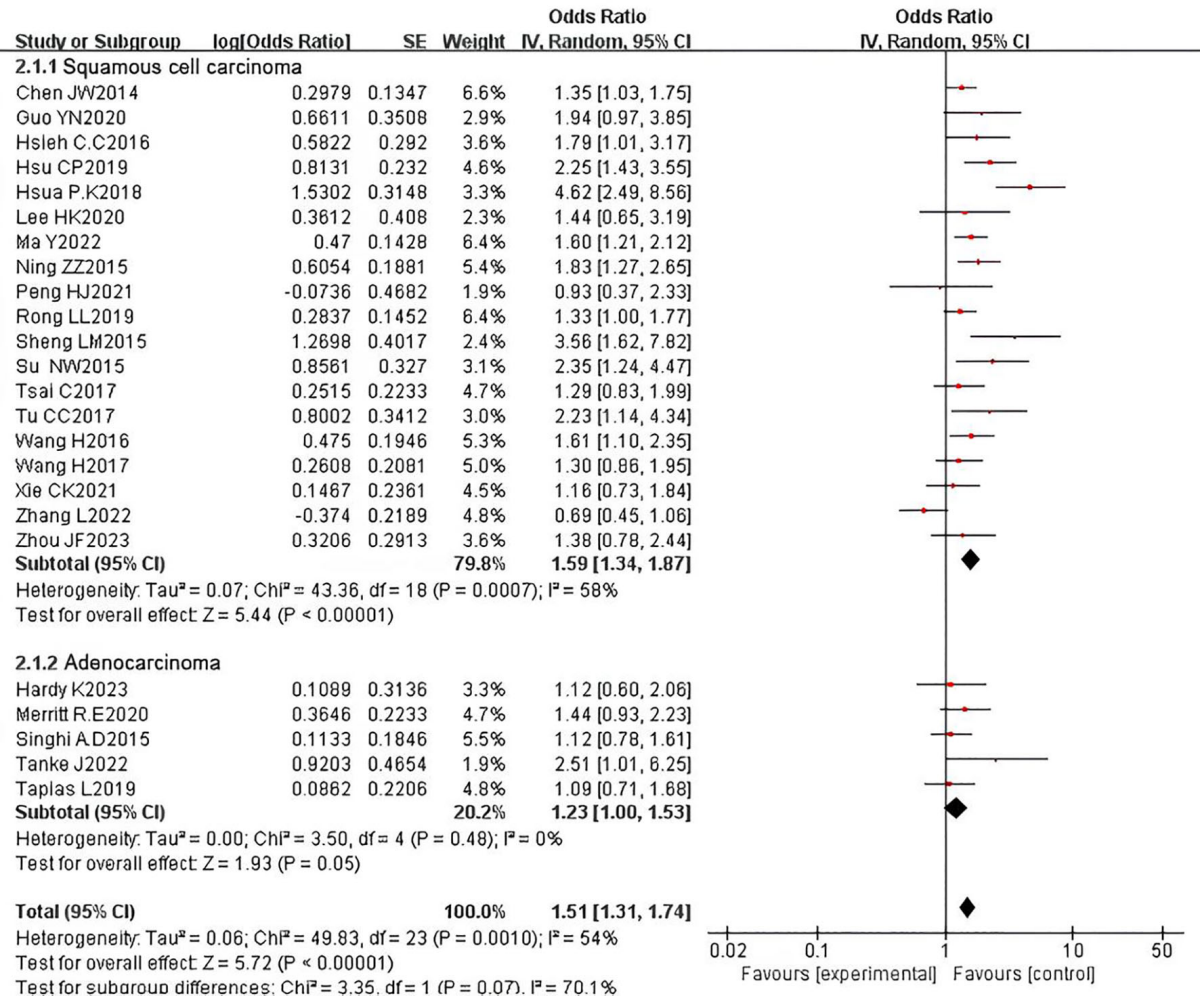
Subgroup analysis forest plot for overall survival (OS) by histological subtype: esophageal squamous cell carcinoma (ESCC) vs. adenocarcinoma (AC).

**Figure 9:**
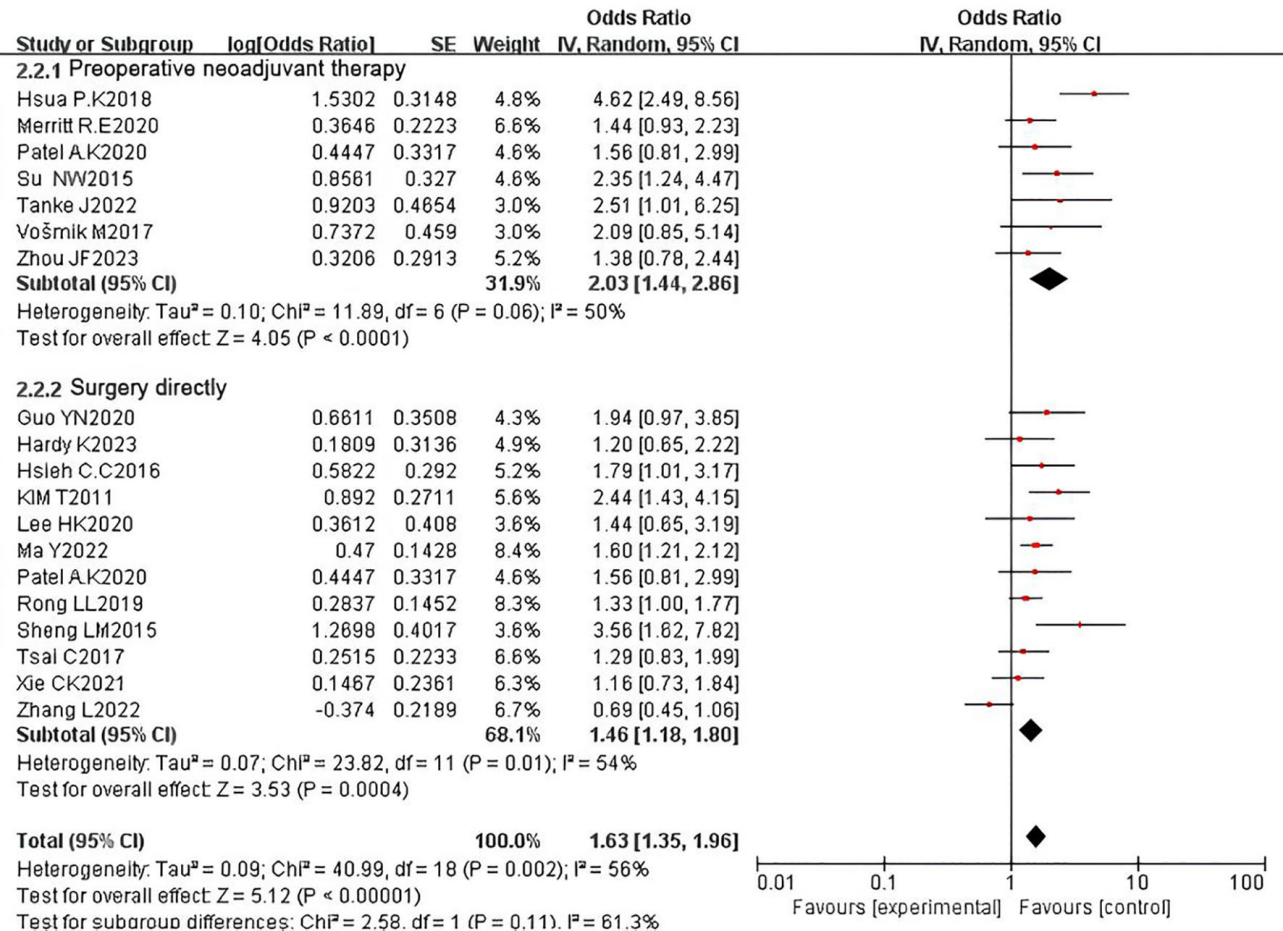
Subgroup analysis forest plot for disease-free survival (DFS) by histological subtype: esophageal squamous cell carcinoma (ESCC) vs. adenocarcinoma (AC).

To explore the prognostic role of PNI in patients with esophageal cancer of different histological types, we conducted subgroup analyses on studies that exclusively included ESCC or adenocarcinoma (AC) and reported the correlation between PNI and OS. In the ESCC subgroup analysis, which included 18 studies with 4,262 patients, PNI was identified as a prognostic factor for adverse OS (HR = 1.62, 95% CI: 1.35-1.94, P < 0.00001). In contrast, in the AC subgroup analysis, which included 5 studies with 1,480 patients, PNI could not be considered a prognostic factor for adverse OS (HR = 1.23, 95% CI: 1.00-1.53, P = 0.05). Significant heterogeneity was observed in the ESCC subgroup (P = 0.0006, I² = 60%), while no heterogeneity was detected in the AC subgroup (P = 0.48, I² = 0%) (**Figure 8**). We also performed subgroup analyses for DFS and obtained similar results. In the ESCC subgroup, the pooled HR was 1.28 (95% CI: 1.03-1.59, P = 0.03), indicating that PNI is a prognostic factor for adverse DFS in ESCC. However, in the AC subgroup, the pooled HR showed no statistical significance (HR = 1.65, 95% CI: 0.95-2.87, P = 0.08). No heterogeneity was observed in either subgroup (ESCC: P = 0.77, I² = 0%; AC: P = 0.35, I² = 0%) (**Figure 9**).

**Figure 10:**
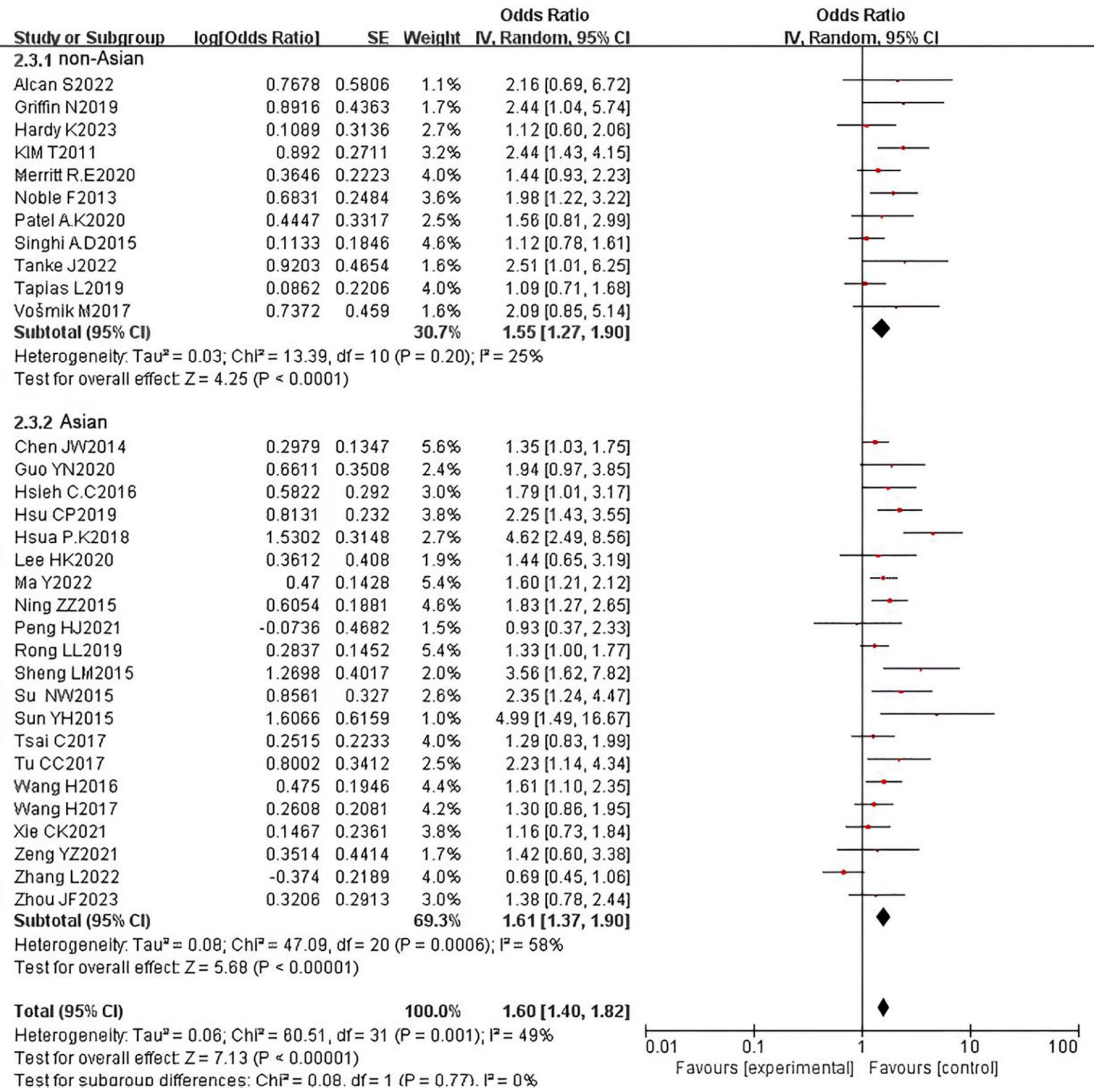
Subgroup analysis forest plot for overall survival (OS) by neoadjuvant therapy status: received vs. not received.

**Figure 11:**
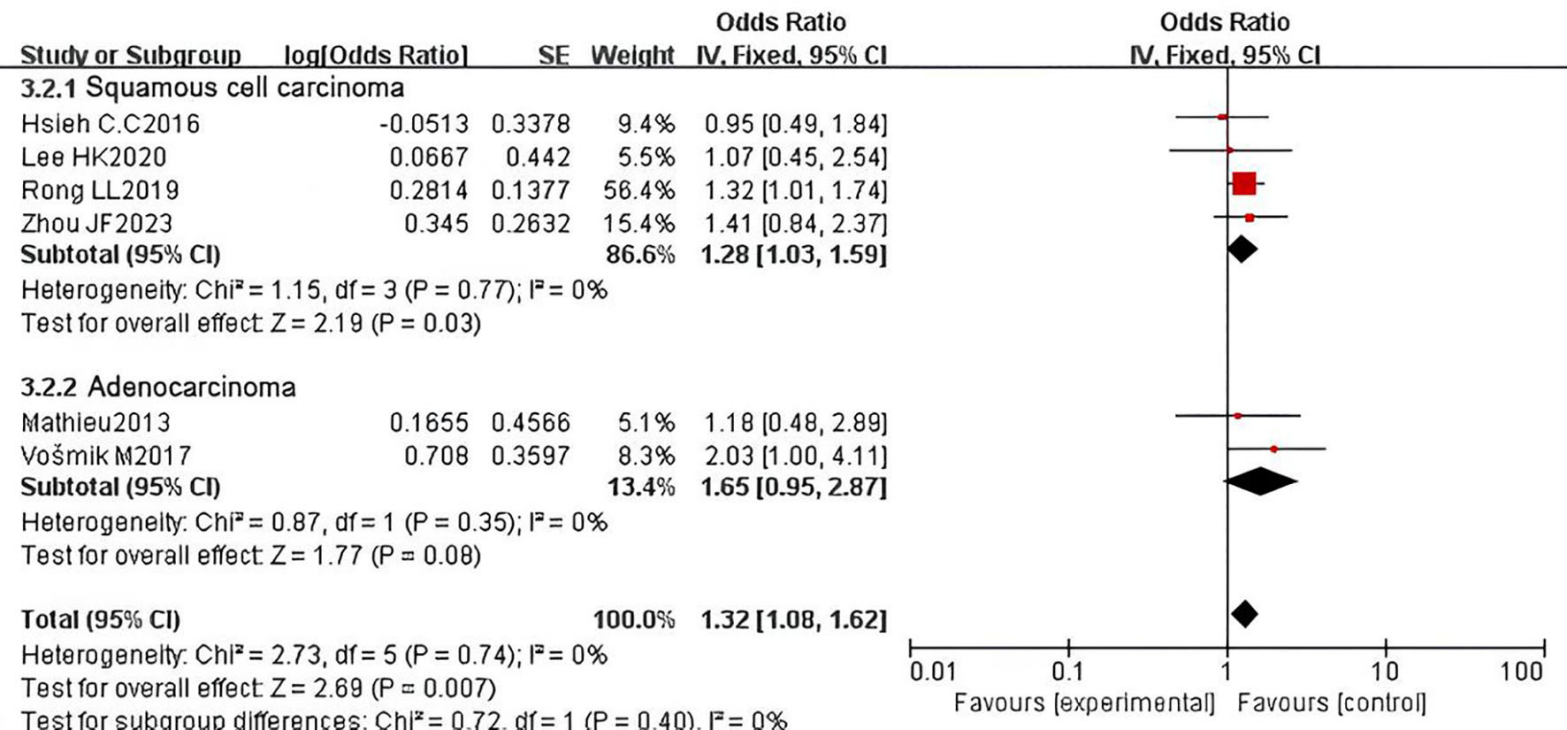
Subgroup analysis forest plot for disease-free survival (DFS) by neoadjuvant therapy status: received vs. not received.

We also conducted subgroup analyses based on whether patients received preoperative neoadjuvant therapy and reported OS outcomes. Among the studies, seven included 1,318 patients with esophageal cancer who received preoperative neoadjuvant therapy, while 12 studies included 3,971 patients who underwent surgery directly. The pooled HR revealed that PNI was a prognostic factor for adverse OS regardless of whether neoadjuvant therapy was administered (neoadjuvant subgroup: HR = 2.03, 95% CI: 1.44-2.86, P < 0.0001; surgery subgroup: HR = 1.49, 95% CI: 1.18-1.87, P = 0.0008). Significant heterogeneity was observed in both subgroups (neoadjuvant subgroup: P = 0.06, I² = 50%; surgery subgroup: P = 0.01, I² = 53%) (**Figure 10**). Similarly, we performed subgroup analyses for studies reporting DFS. The pooled results indicated that PNI was a prognostic factor for adverse DFS regardless of whether neoadjuvant therapy was administered (neoadjuvant subgroup: HR = 1.58, 95% CI: 1.09-2.28, P = 0.02; surgery subgroup: HR = 1.38, 95% CI: 1.19-1.59, P < 0.0001). No heterogeneity was detected in either subgroup (neoadjuvant subgroup: P = 0.71, I² = 0%; surgery subgroup: P = 0.71, I² = 0%) (**Figure 11**).

**Figure 12:**
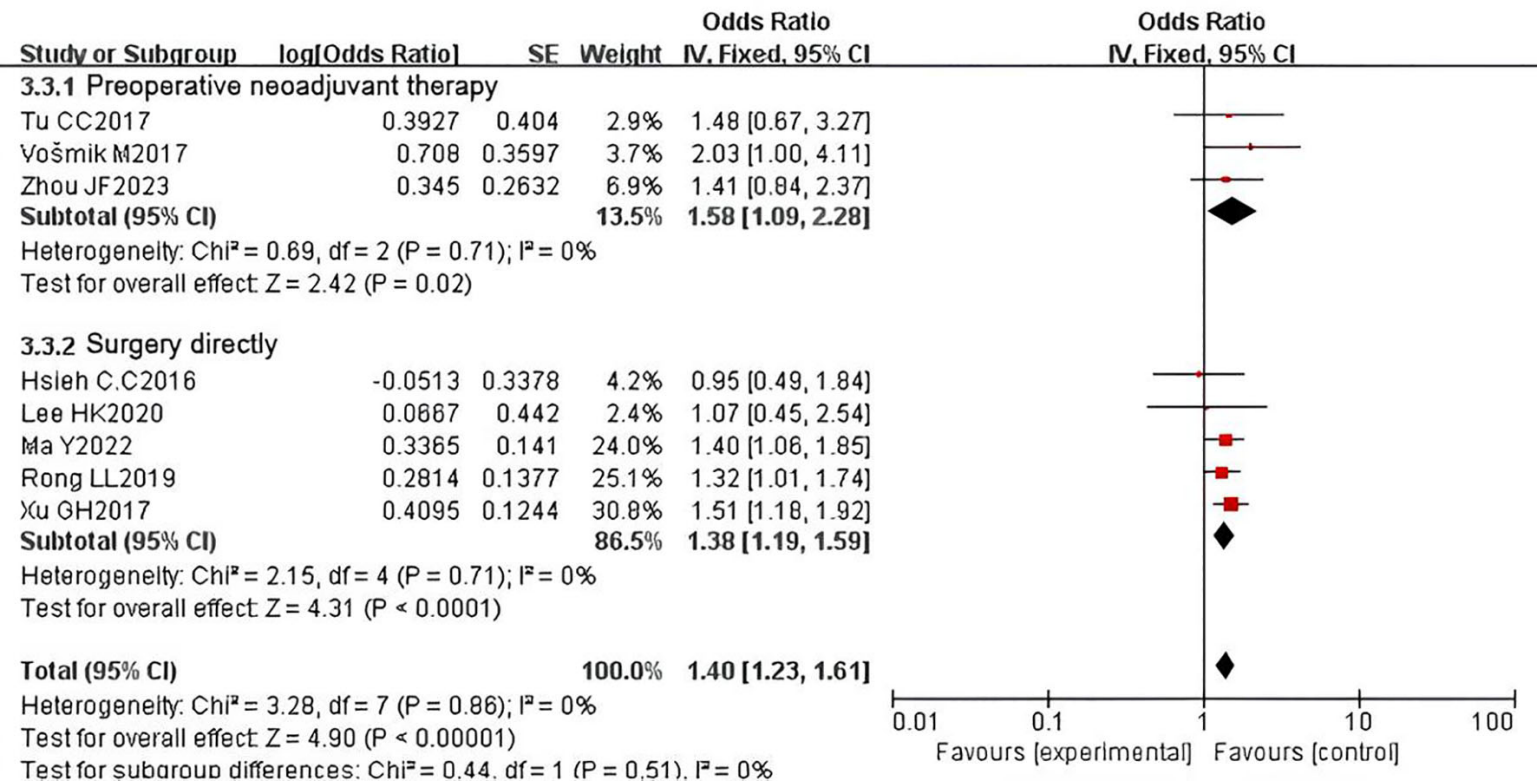
Subgroup analysis forest plot for overall survival (OS) by ethnicity: Asian vs. non-Asian.

**Figure 13:**
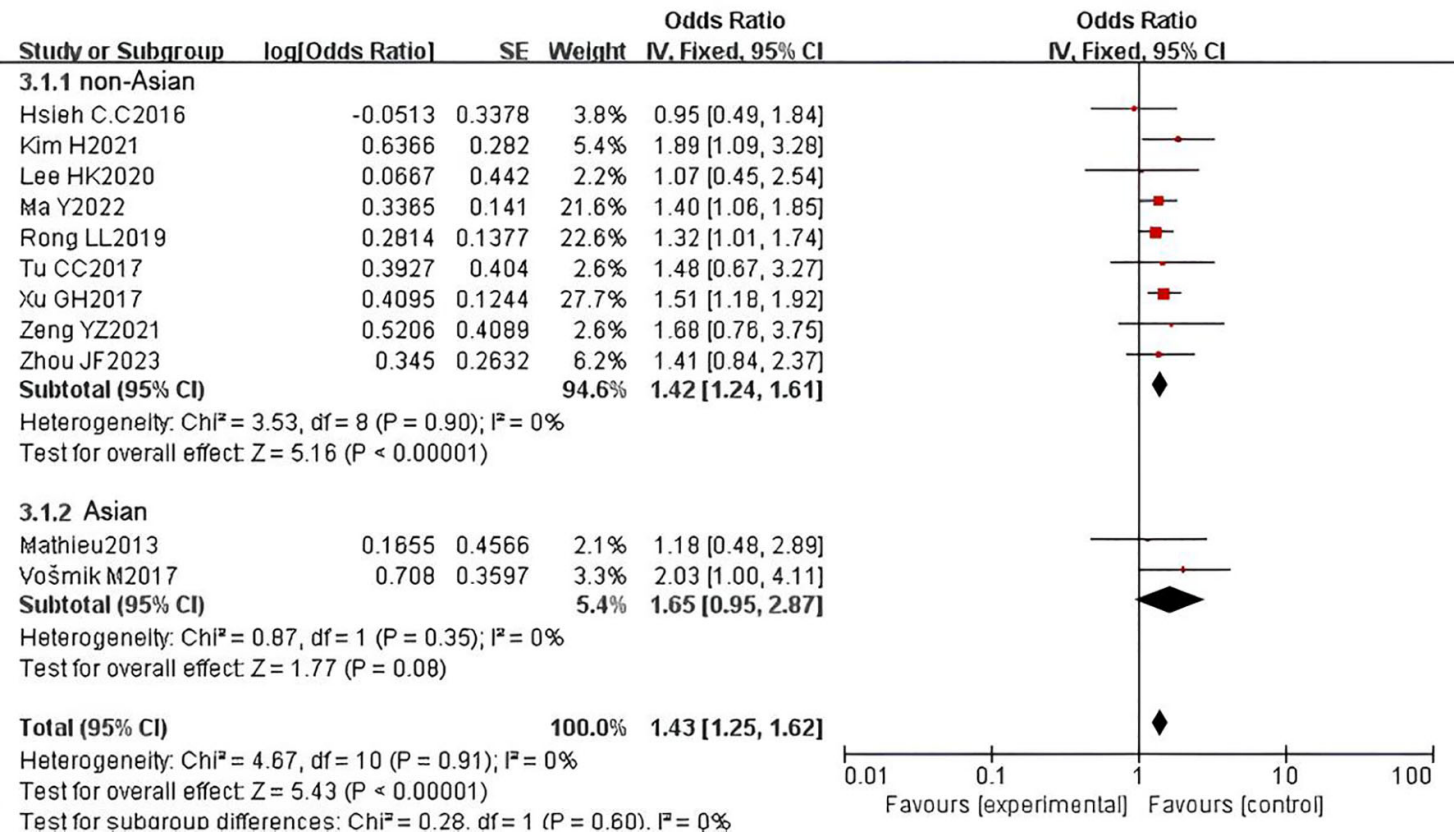
Subgroup analysis forest plot for disease-free survival (DFS) by ethnicity: Asian vs. non-Asian.

We further conducted subgroup analyses based on ethnicity, comparing Asian and non-Asian cohorts. The results indicated that ethnicity does not influence the prognostic role of PNI in OS. Specifically, PNI remained a significant prognostic factor for adverse OS in both Asian (HR = 1.64, 95% CI: 1.38-1.95, P < 0.000001) and non-Asian (HR = 1.55, 95% CI: 1.27-1.90, P < 0.001) populations. However, significant heterogeneity was observed in the Asian subgroup (P = 0.0008, I² = 57%), while mild heterogeneity was present in the non-Asian subgroup (P = 0.20, I² = 25%) (**Figure 12**). In the analysis of DFS, the pooled HR revealed that PNI is a prognostic factor for adverse DFS in the Asian subgroup (HR = 1.42, 95% CI: 1.24-1.61, P < 0.00001), but not in the non-Asian subgroup (HR = 1.43, 95% CI: 1.25-1.62, P = 0.08). No heterogeneity was detected in either the Asian (P = 0.90, I² = 0%) or non-Asian (P = 0.91, I² = 0%) subgroups (**Figure 13**).

### Publication bias

This study did not exhibit significant publication bias or selection bias.

## Discussion

PNI has been previously established as a poor prognostic factor in esophageal cancer patients. Thirty-nine studies from various countries have confirmed the prognostic significance of PNI in esophageal cancer patients. PNI was identified as an independent prognostic factor for OS, DFS, and distant metastasis, but not for recurrence.

However, recent large-scale studies have challenged this view (12–14). This meta-analysis updates the prognostic role of PNI in esophageal cancer. Unlike previous meta-analyses, the study comprehensively evaluated the prognostic implications of PNI in esophageal cancer, extending beyond OS to include DFS and recurrence/metastasis outcomes—dimensions not systematically explored in earlier research. Considering the established correlations between PNI and heterogeneous pathological characteristics, we preemptively addressed potential homogeneity or collinearity biases by exclusively pooling adjusted HRs from multivariate survival analyses. This methodological approach minimized confounding effects and strengthened the validity of our conclusions.

Subgroup analyses were further performed based on histopathological subtype (ESCC vs. AC), administration of preoperative neoadjuvant therapy, and ethnicity to delineate the prognostic role of PNI across clinically relevant subgroups. Notably, the prognostic significance of PNI exhibited marked heterogeneity among these subgroups. In ESCC, PNI independently predicted both OS and DFS, whereas no statistically significant associations were observed in adenocarcinoma patients. The prognostic utility of PNI for OS and DFS persisted irrespective of preoperative neoadjuvant therapy status. Furthermore, while PNI demonstrated prognostic relevance in Asian populations, its association with DFS lacked statistical significance in non-Asian cohorts.

Previous studies have suggested that PNI is more commonly observed in adenocarcinoma (55). However, a recent meta-analysis indicated no statistically significant difference in the distribution of PNI between esophageal adenocarcinoma and squamous carcinoma. A meta-analysis by Hsu et al. demonstrated that PNI could predict OS in both ESCC and AC, which aligns with our preliminary findings. Previous studies have suggested that PNI is associated with the prognosis of both squamous cell carcinoma and adenocarcinoma. However, after conducting further subgroup analysis in our study, we found that PNI is not an adverse prognostic factor for OS or DFS in adenocarcinoma, which is inconsistent with prior clinical studies (35,56). Earlier research proposed that preoperative neoadjuvant therapy might influence PNI status in patients (56), but our pooled analysis of HR values from multiple studies showed that neoadjuvant therapy did not affect the prognostic role of PNI. Notably, our methodology—statistically synthesizing HR values across studies, analyzing OS and DFS separately, and conducting detailed subgroup analyses based on pathological characteristics—provides more robust and convincing evidence. Nonetheless, large-sample studies are warranted to validate these conclusions.

Asia is often referred to as the “esophageal cancer belt” due to its distinct epidemiological and histological characteristics compared to non-Asian regions (57–60). Previous meta-analyses suggested no racial differences in the prognostic role of PNI across ethnic subgroups. However, in our ethnicity-based subgroup analysis, PNI demonstrated prognostic significance for OS in both Asian and non-Asian esophageal cancer patients. In contrast, no association was observed between PNI status and DFS in non-Asian patients. We hypothesize that this discrepancy may stem from the predominance of adenocarcinoma in non-Asian esophageal cancer populations, aligning with our earlier subgroup findings. Nevertheless, we acknowledge potential bias due to the limited number of pooled DFS-focused studies in non-Asian cohorts.

We must also acknowledge the limitations of these studies. Notably, significant heterogeneity was observed in the analyses involving OS. When exploring the correlation between PNI and OS, funnel plots revealed evident heterogeneity. Through stepwise exclusion of studies, we identified the source of heterogeneity: two large-scale studies contributing to this variability. Further analysis of these two heterogeneous studies—both with substantial sample sizes—suggested that their scale might underlie the observed discrepancies. After excluding these studies, heterogeneity was markedly reduced. Although one of these studies, encompassing 794 cases, concluded that PNI was not a prognostic factor for OS, its exclusion did not alter our final conclusions, prompting us to retain it in the analysis. Additionally, all included cohorts were retrospective, which inherently introduces potential heterogeneity. While we employed the Newcastle-Ottawa Scale (NOS) for quality assessment in this meta-analysis, some scholars argue that its application in evidence evaluation and meta-analyses may lead to highly subjective outcomes (61).

Perineural invasion represents a unique pathological micrometastatic process, and its underlying molecular mechanisms require further investigation. Our study updates the prognostic role of PNI in esophageal cancer, particularly highlighting its inconsistent prognostic significance across different patient subgroups. Furthermore, our findings suggest that PNI should be incorporated into prognostic risk stratification for distinct esophageal cancer subgroups. However, due to the anatomical distribution of nerves in esophageal cancer, PNI status cannot currently be prospectively assessed preoperatively. This limitation precludes prospective trials evaluating neoadjuvant or postoperative adjuvant therapies tailored to PNI status. Nonetheless, multiple studies have confirmed that postoperative adjuvant therapy improves survival rates in PNI-positive esophageal cancer patients. We anticipate that advancements in endoscopic technology may enable preoperative determination of PNI status in the future. Such progress would not only enhance surgical and therapeutic planning accuracy, thereby improving patient survival and prognosis, but also drive the development of precision medicine.
